# Antigen persistence after antibody-drug conjugates in urothelial carcinoma: implications for CAR-cell target selection

**DOI:** 10.1016/j.neo.2026.101383

**Published:** 2026-10-17

**Authors:** Jiayi Liu, Shaoqiang Xing, Qian Liu

**Affiliations:** a The First Central Clinical College of Tianjin Medical University, No. 24 Fukang Road, Nankai District, Tianjin 300192, China; b Department of Urology, Tianjin First Central Hospital, No. 24 Fukang Road, Nankai District, Tianjin 300192, China

**Keywords:** Urothelial carcinoma, Antibody-drug conjugate, Enfortumab vedotin, NECTIN4, CAR T cell, Drug resistance

## Abstract

Antibody-drug conjugates (ADCs) and chimeric antigen receptor (CAR)-engineered cells can converge on the same surface antigens in urothelial carcinoma (UC) yet depend on distinct biological steps. This creates a central uncertainty after ADC progression: whether failure reflects loss of membrane antigen or a downstream resistance mechanism that a CAR might bypass. We synthesize paired human tissue, molecular-imaging, ADC-resistance and UC-specific CAR-cell studies to examine whether an antigen targeted by a failed ADC remains suitable for cellular therapy. NECTIN4 after enfortumab vedotin (EV) provides the most direct evidence. Membranous expression often decreases between primary and metastatic UC, and serial observations document lesion-specific loss. However, selected post-EV biopsies and resistant models retain NECTIN4; in those models, NECTIN4 CAR T cells remain active. Transporter and payload-resistance studies further support target-independent failure. Evidence for HER2 and TROP2 is less direct but suggests that alternative antigens may remain available when NECTIN4 declines. We therefore propose a resistance-state framework: same-target CAR development requires recent evidence of accessible membrane antigen; target loss or marked heterogeneity favors target switching or multi-target strategies; and unresolved states require biomarker-rich testing. Current evidence does not support a clinical treatment sequence. Instead, it defines the sampling, biomarker and correlative-study requirements needed to make post-ADC CAR trials interpretable.

## Highlights

• ADC failure does not by itself determine CAR-target suitability.
• After enfortumab vedotin, NECTIN4 may be lost or retained.
• Resistance mechanism—not progression alone—guides same-target CAR reuse.
• When feasible, recent lesion reassessment should inform CAR-trial design.
• Multi-target strategies may be needed when treatment-shaped heterogeneity persists.

## Introduction

Enfortumab vedotin (EV) plus pembrolizumab has moved NECTIN4-directed therapy into the first-line treatment of locally advanced or metastatic urothelial carcinoma (UC) after demonstrating superior survival to platinum-based chemotherapy in EV-302 [1]. Candidates for experimental cellular therapy will therefore increasingly have tumours already exposed to selective pressure against a therapeutically relevant surface antigen. Target selection for chimeric antigen receptor (CAR)-engineered cells can no longer rely on expression in treatment-naive primary tumours.

ADCs and CAR-engineered cells can recognise the same antigen yet fail for different reasons. An ADC must bind, internalise and process its target, release an active payload, and overcome mechanisms that expel or tolerate that payload. A CAR requires accessible surface antigen and a productive immune synapse, but not linker processing or payload susceptibility. This distinction also applies to T-cell engagers [2]. Thus, radiographic progression cannot distinguish target-dependent from target-independent resistance (Fig. 1).

**Fig. 1:**
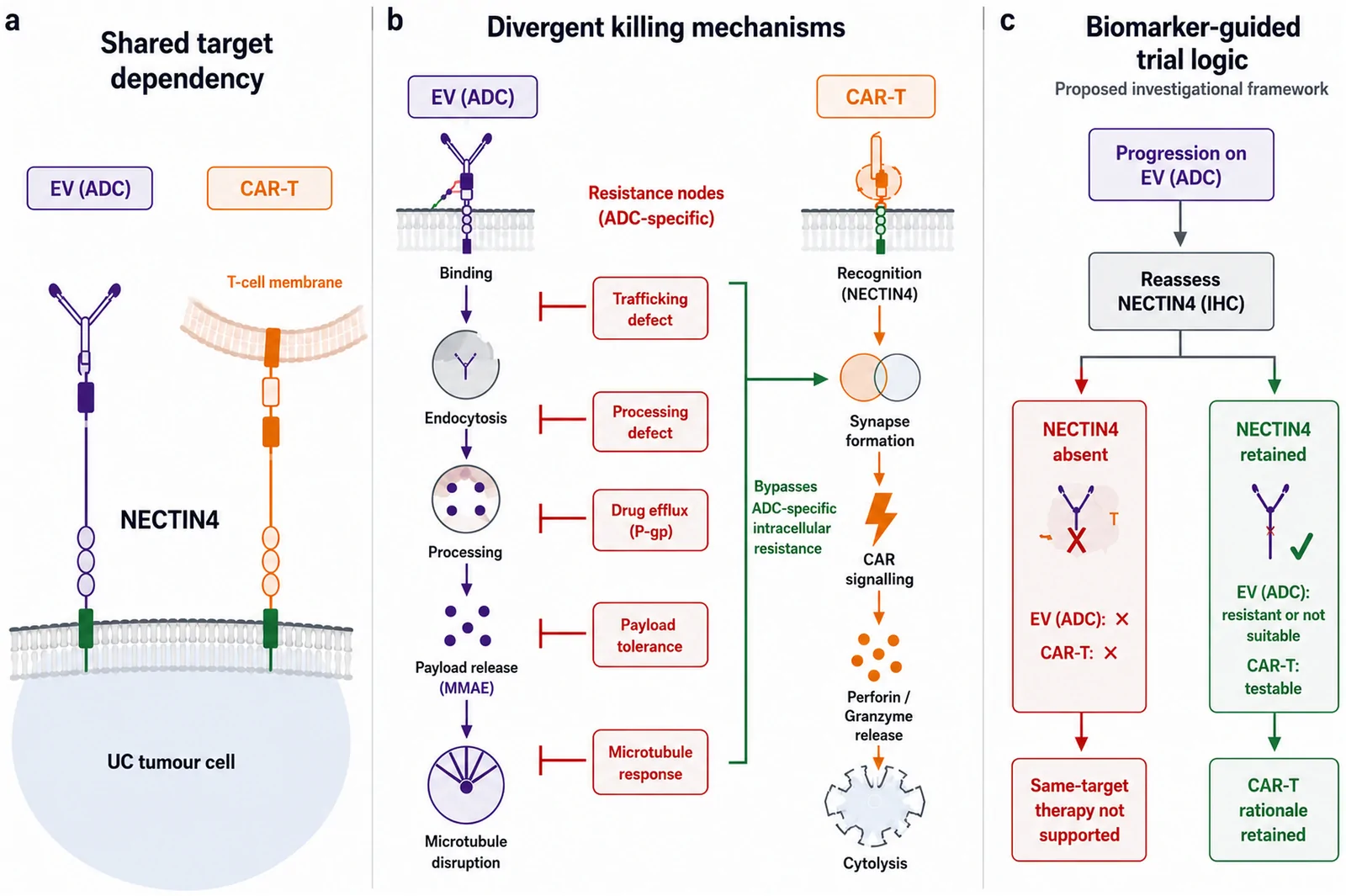
**| Shared NECTIN4 dependency and divergent EV and CAR-T killing mechanisms.** a EV and a NECTIN4-directed CAR-T cell both require sufficient, accessible NECTIN4 on the tumour-cell surface. b After binding, EV depends on endocytosis and trafficking, intracellular processing, release and retention of monomethyl auristatin E (MMAE), and a susceptible microtubule response. Defects in trafficking or processing, P-glycoprotein (P-gp)-mediated efflux, payload tolerance and altered microtubule response are shown as alternative EV/ADC-specific resistance nodes and need not occur concurrently. CAR-T cells instead require NECTIN4 recognition, immune-synapse formation and CAR signalling, followed by perforin/granzyme release and cytolysis. CAR-T cells may therefore bypass EV-specific intracellular resistance downstream of target recognition but do not bypass the requirement for sufficient, accessible surface NECTIN4. c Proposed investigational framework after progression on EV. Absence of membrane NECTIN4 removes the direct recognition basis for both EV and an unmodified same-target CAR-T product. Retained NECTIN4 after EV progression preserves a rationale for CAR-T trial testing but does not establish efficacy. The binary branches are conceptual: target density, heterogeneity, epitope accessibility, sampling and product-specific activation thresholds remain unresolved. This is an original conceptual synthesis based on 3 and [4], [5], [6], [7], [8], [9], [10],^11^,^12^.

Available human evidence also challenges the assumption that a target measured once remains stable. NECTIN4 expression differs across molecular subtypes, with enrichment in luminal tumours and lower expression in several basal or neuroendocrine-like contexts [13]. In 137 paired primary and metastatic UC specimens, membranous NECTIN4 decreased significantly during metastatic spread, and 39.4% of metastases lacked membranous expression [14]. More recent matched-specimen cohorts similarly show variation across transurethral resection, cystectomy, lymph-node and distant metastatic samples [[15], [16], [17]]. A rapid-autopsy series further demonstrated that membrane and cytoplasmic localisation can differ across metastatic sites and variant histologies [18]. Variant histology itself may add actionable heterogeneity: small-cell UC had much lower NECTIN4 and TROP2 expression than conventional UC in one comparative cohort, whereas recent multi-omic work identified a CA125-positive state and preclinical TM4SF1 targetability in aggressive bladder-cancer variants [19,20]. Conversely, a study of 17 matched biopsies obtained before and after EV found that NECTIN4 increased in 9 pairs and was not globally depleted at resistance; EV-resistant models retaining NECTIN4 remained susceptible to NECTIN4 CAR T cells [3]. Serial NECTIN4-targeted positron-emission tomography has likewise visualised progressive lesions with intense target signal during EV-based treatment [21]. Thus, both target loss and target persistence are biologically credible after progression.

Existing reviews have addressed ADC resistance in UC, HER2-directed treatment, and CAR-cell therapy in bladder or genitourinary cancer separately [[22], [23], [24], [25]]. Their intersection remains unresolved: after an ADC has failed, which resistance states preserve a rationale for reusing the same antigen through a CAR, which require target switching, and which cannot be interpreted without new tissue or imaging? We address this question through a reproducible critical review of human antigen-dynamics studies, ADC-resistance mechanisms and functional CAR evidence. The objective is not to present CAR-cell therapy as established post-ADC treatment, but to define a mechanism-based framework for target selection and correlative testing in future trials.

## Evidence identification and synthesis

We searched MEDLINE/PubMed using eight prespecified query families covering UC, the principal ADC targets and products, ADC resistance, CAR-engineered cells, target reassessment and antigen escape. Embase, Scopus and Web of Science Core Collection were searched on 27 July 2026 with aligned disease, platform/target and resistance/target-state concept blocks. OpenAlex, Crossref, publisher records and PubMed Central supported supplementary discovery and citation verification. The four bibliographic databases yielded 5,016 source records and 3,601 unique candidates after deduplication by DOI, PMID and normalised title–author combinations.

Automated concept triage reduced the deliberately broad retrieval, after which potentially relevant records underwent manual assessment and every cited study underwent claim-level verification. Jiayi Liu performed the primary screen. Shaoqiang Xing independently verified all included studies and a sample of exclusions, and disagreements were resolved by consensus with Qian Liu.

We prioritised human paired or serial specimens, post-ADC tissue, resistant models with direct target measurement, and CAR experiments conducted after ADC resistance. Treatment-naive expression studies were treated as contextual evidence and were not used to establish post-treatment persistence. Reviews defined intellectual context and overlap but did not substitute for primary evidence. Heterogeneity in specimen timing, assays, resistance definitions and model systems precluded quantitative meta-analysis. The complete search protocol, strategies, export audit and screening decisions accompany the manuscript as Supplementary Files 1 and 2.

## The same antigen does not imply the same therapeutic dependency

### Shared requirement: accessible membrane antigen

ADCs and CARs require accessible tumour-cell surface epitopes. Gene amplification, transcript abundance and total protein expression are incomplete proxies for membrane density. NECTIN4 amplification correlates with membrane staining and EV response [26]. A recent cohort combined membranous NECTIN4 scoring with amplification to refine EV response prediction [27], but neither assay defines a CAR activation threshold. The earlier paired study used a knockout-validated H-score [14]. Diagnostic IHC may not recognise the therapeutic antibody or CAR epitope. Cytoplasmic staining, bulk RNA and unqualified genomic alterations should therefore not be treated as equivalent evidence for CAR-cell selection. Supplementary Table S1 separates the evidentiary maturity of these assays and records the assay variables that must be reported.

Surface abundance is continuous rather than binary. NECTIN4 CAR T-cell activity increased in higher-expressing UC models, and PPARγ activation increased NECTIN4 and CAR activity [3]. No validated UC-specific density threshold separates likely CAR-responsive from non-responsive tumours. The lymphoma observation that CD19 immunohistochemistry alone did not predict response to a CD19 ADC is not a UC rule, but reinforces that pathology cannot substitute for quantitative, product-relevant surface-target assessment [28].

### ADC-specific dependencies

After surface binding, an ADC depends on several steps that are not required for CAR signalling. These include receptor-mediated internalisation, intracellular trafficking, linker processing, payload release and cellular sensitivity to the payload [4,5]. Experimental modulation of sialylation in bladder-cancer models illustrates how these steps can interact: increased sialylation was associated with lower NECTIN4, reduced EV endocytosis and lower treatment activity, whereas sialylation inhibition increased both target abundance and internalisation [6]. More direct work has identified endocytic evasion and extracellular-vesicle disposal of ADC–target complexes as a resistance mechanism in NECTIN4-directed therapy [7]. Although antigen and internalisation may change together, they remain analytically distinct variables.

Payload resistance provides a clearer example of potential mechanistic divergence. EV delivers monomethyl auristatin E (MMAE). MMAE-resistant bladder-cancer models showed DPP4-associated changes and could be resensitised through DPP4 inhibition without establishing loss of an ADC target [8]. Similarly, clinical and translational studies have associated ABCB1/P-glycoprotein, ABCC1/MRP1 and ABCG2/BCRP expression with shorter EV benefit or acquired EV resistance [9,10]. Tumour-cell efflux can lower intracellular payload exposure, but it should not directly prevent a CAR T cell from recognising an intact extracellular epitope.

Payload choice can change the interpretation of same-target failure. EV plus the TROP2-directed SN-38 ADC sacituzumab govitecan produced responses in the small, non-randomised DAD phase I study [29]. Preliminary NEXUS-01 results for the NECTIN4/topoisomerase-I ADC LY4052031 also included activity in EV-pretreated UC [30]. These studies neither establish an ADC-to-CAR sequence nor show that DNA-repair signatures select MMAE versus topoisomerase-I payloads. They do require prior EV exposure to be described by target, binder, linker and payload.

### CAR-specific dependencies

CAR activity replaces ADC internalisation and payload delivery with immune-synapse formation, activation, expansion and serial cytotoxicity. Retention of an antigen is therefore necessary but not sufficient. Low density, heterogeneous distribution, limited trafficking of effector cells, suppressive tumour microenvironment, T-cell dysfunction and normal-tissue expression can all restrict efficacy or safety [11]. In UC, immune exclusion associated with PPARγ/RXRα activation provides a specific reason not to interpret an antigen-retaining tumour as CAR-permissive without parallel immune-contexture assessment [31]. On-target, off-tumour toxicity is especially consequential in solid tumours, making quantitative normal-tissue expression and product-specific activation thresholds part of target validation rather than a late safety exercise [12]. Moreover, pharmacologically increasing a target in tumour cells may also increase expression in normal tissues. Antigen modulation should consequently be treated as a therapeutic-window experiment, not simply as a sensitisation strategy.

The most informative evidence tests both modalities in the same resistant state. RT112 cells exposed to EV for nine months had an approximately tenfold higher EV half-maximal inhibitory concentration while retaining total and surface NECTIN4; they and resistant xenografts remained CAR-sensitive [3]. A second model with moderately reduced target also remained CAR-sensitive. This supports mechanistic non-equivalence in selected models, not all EV-resistant UC.

## A resistance taxonomy organised by its consequence for CAR

From a CAR-development perspective, ADC resistance separates into consequential states rather than a single post-treatment category. Antigen loss or reduction directly weakens same-target recognition; target-retaining ADC-specific resistance preserves a mechanistic rationale; and spatial heterogeneity or CAR-specific barriers require mapping or product optimisation rather than a binary target decision (Fig. 2 and Table 2).

**Fig. 2:**
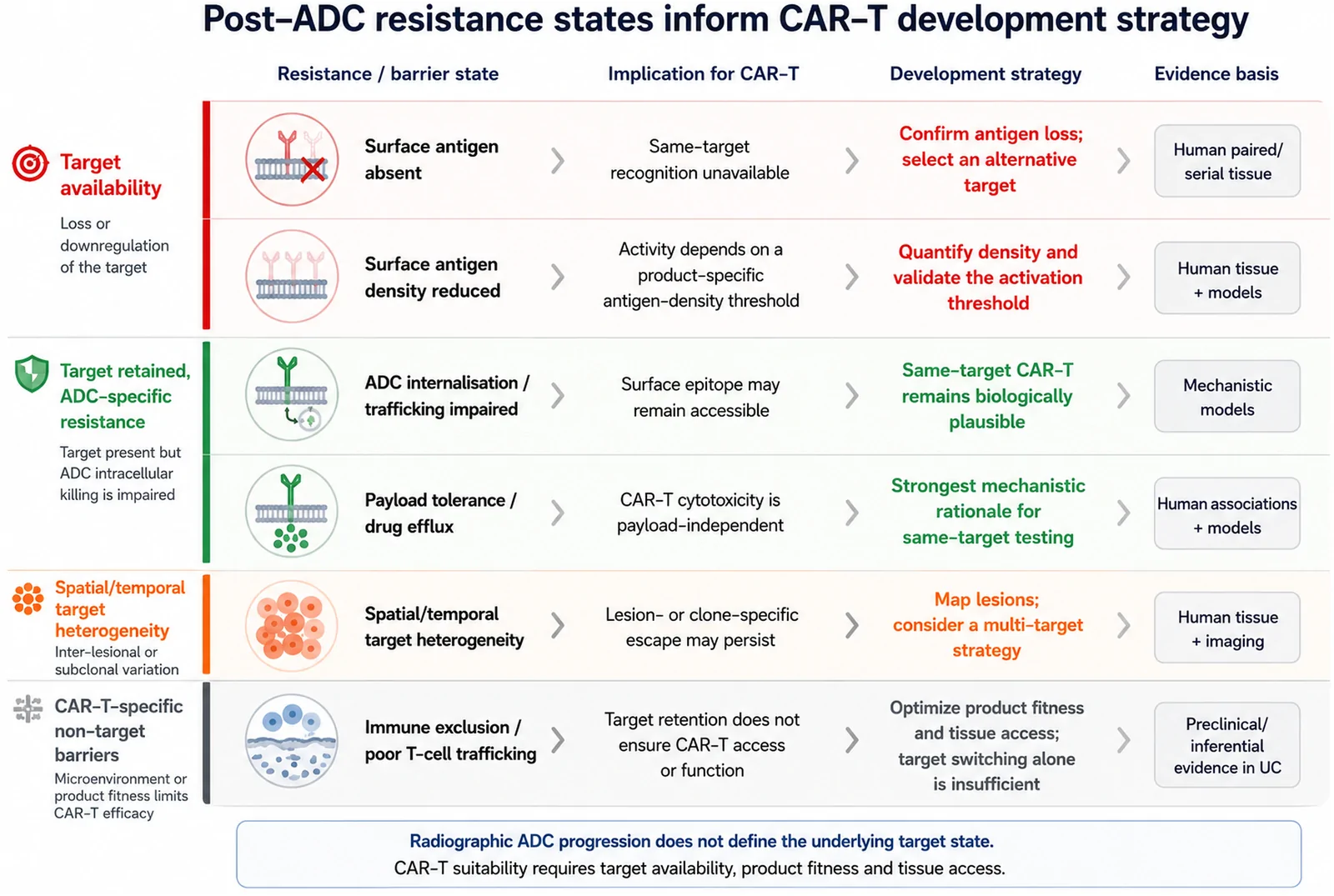
**Post-ADC resistance states inform CAR-T development strategy.** Six post-ADC resistance or barrier states are organised by their consequence for same-target CAR-T development, the corresponding development strategy and the directness of current evidence in urothelial carcinoma. Surface-antigen absence removes the basis for same-target recognition and supports confirmation of antigen loss followed by selection of an alternative target. Reduced antigen density makes activity dependent on a product-specific activation threshold. When the target is retained, impaired ADC internalisation or trafficking, payload tolerance or drug efflux can preserve a biological rationale for same-target CAR-T testing because CAR-T cytotoxicity does not depend on ADC intracellular processing or payload activity. Spatial or temporal heterogeneity supports lesion mapping and consideration of multi-target strategies, whereas immune exclusion or poor T-cell trafficking requires optimisation of product fitness and tissue access rather than target switching alone. Radiographic ADC progression does not define the underlying target state. This is an original evidence framework based on [3], [13], [14], [15], [16], [17], [18], [19], [20], [21],[6], [7], [8], [9], [10] and [32], [33], [34], [35], [36], [37],[38], [39], [40], [41], [42], [43], [44].

### Antigen-dependent resistance

True loss of membrane antigen is the most direct reason to abandon unmodified same-target CAR reuse. NECTIN4 can decline during metastatic evolution, and serial clinical reports describe marked reduction in lesions progressing during EV [14,32]. In one case, decreased Nectin-4 occurred together with increased ABCB1/MDR1, ABCC1/MRP1 and ABCG2/BCRP, illustrating that antigen loss may coexist with other mechanisms [10]. A recent paired cohort found a change in NECTIN4 status between primary and metastatic samples in 33.9% of 56 patients [16]. These observations argue against assigning CAR eligibility from the diagnostic specimen alone. They do not permit a pooled estimate of the proportion of EV-treated patients with post-EV antigen loss: the available studies differ in treatment exposure, lesion timing, assay and definition of loss (Table 1).

**Table 1:** Human evidence for dynamic ADC-associated targets in urothelial carcinoma.

Study	Clinical context	Specimens or patients	Target assessment	Principal finding	Relevance to CAR selection	Limitations
Klümper et al., 2023; PMID 36534531	Metastatic evolution; separate EV-treated cohort	137 paired primary/metastatic specimens; 47 EV-treated patients	Validated membrane-specific NECTIN4 IHC and H-score	39.4% of metastases lacked membranous NECTIN4; low/negative expression correlated with shorter EV PFS	Archival primary tissue may overestimate metastatic CAR target availability	Paired cohort was collected before metastatic therapy, not post-EV
Chang et al., 2025; PMID 40931013	Acquired EV resistance	17 matched pre/post-EV biopsies plus resistant models	NECTIN4 IHC and surface assays	NECTIN4 increased in 9/17 matched biopsies; resistant models often retained target	Supports same-target CAR testing when recent tissue confirms retention	Small cohort; direct CAR evidence remains preclinical
ABC-transporter cohort, 2025; PMID 39877564	EV-treated mUC	20 patients	Nectin-4 and ABC-transporter IHC	High ABC-transporter expression and lower Nectin-4 associated with poorer EV outcomes	Target and payload-efflux mechanisms should be assessed separately	Small retrospective cohort
Serial EV-resistance case, 2024; PMID 38440718	Lesions progressing on EV	1 patient	Nectin-4, MDR1, MRP1 and BCRP IHC	Target decreased while multiple efflux transporters increased	Demonstrates a mixed resistance phenotype	Case-level evidence
Matched bladder triplets, 2026; PMID 41986174	TURBT, cystectomy and lymphadenectomy	27 patients	NECTIN4 IHC; NECTIN4, TACSTD2 and ERBB2 expression	Target levels varied substantially across specimen types	Trial eligibility should document which lesion and timepoint were tested	Not post-ADC
Site-specific advanced UC study, 2026; PMID 42422333	Primary and metastatic UC	139 primary; 56 paired metastatic	Membrane NECTIN4 IHC	NECTIN4 status changed in 33.9% of pairs; patterns differed by bladder versus upper tract origin	Supports site-specific rather than diagnosis-level target assignment	Not specifically post-EV
HER2 genomic/functional study, 2025; PMID 41422272	Primary and metastatic UC; HER2-directed treatment context	2,035 profiled UC tumours; 30 matched primary–metastatic pairs; patient-derived models	ERBB2 sequencing, protein assays and functional ADC testing	ERBB2 status was discordant in 12/30 matched pairs; some models diverged from source tumours	Supports recent assay-specific reassessment and cautions against genomic status as a membrane-target surrogate	Mostly genomic rather than membrane-level dynamics; not a post-ADC CAR cohort
Multi-target landscape, 2026; PMID 42488113	UC progression and anatomical heterogeneity	118 specimens; smaller paired subsets	NECTIN4, HER2 and TROP2 IHC	56.8% showed multi-target co-expression; NECTIN4 tended to decline in metastases	Supports investigation of switching and OR-gated designs	No post-ADC cohort or CAR experiment
NECTIN4 PET proof of principle, 2026; PMID 40847665	Before EV plus pembrolizumab	10 patients	Gallium-68-N188 PET/CT with available IHC	Frequent within-patient inter-lesional heterogeneity	Whole-body imaging may complement a single biopsy in trials	Pretreatment and investigational
Serial NECTIN4 PET case, 2026; PMID 42257446	Progression during EV plus pembrolizumab	1 patient	Gallium-68-NECT-224 PET/CT	New progressive lesions retained intense target signal	Clinical progression does not prove target loss	Single case; no CAR threshold
Serial salvage-surgery case, 2026; PMID 41403786	Isolated progression after prolonged EV	1 patient	Pre/post-treatment Nectin-4 IHC	Marked target decline in the progressing primary tumour	Target loss can be lesion-specific during treatment	Single case

**Interpretation rule:** “CAR relevance” is a trial-development implication and not a treatment recommendation. Membrane IHC and PET uptake have not been validated as interchangeable or as CAR eligibility thresholds.

**Table 2:** ADC-resistance mechanisms and their implications for CAR-cell development.

Resistance state	ADC consequence	Expected same-target CAR consequence	Evidence in UC	Trial-development response
Loss of membrane antigen	Reduced ADC binding and delivery	Likely loss of CAR recognition	Paired metastatic NECTIN4 loss; serial EV cases	Retest a recent lesion; switch target or use an appropriate multi-target design
Reduced but persistent antigen density	Lower ADC delivery; effect depends on payload and bystander activity	CAR activity may fall below an activation threshold	Chang et al. showed stronger CAR activity in higher expressors	Quantify membrane density; avoid simple positive/negative classification
Assay–binder uncertainty	A diagnostic signal may not establish recognition by the therapeutic ADC binder	A diagnostic signal may not establish recognition by the CAR binder	UC studies use heterogeneous immunohistochemistry reagents and scoring systems	Report clone, platform, scoring, membrane restriction and evidence of epitope compatibility; use quantitative fresh-tissue assays where feasible
Target retained with payload resistance	ADC binding can remain intact but cytotoxic payload fails	CAR recognition may remain intact	MMAE-resistant models; EV-resistant NECTIN4-retaining models	Same-target CAR is biologically plausible but requires direct functional testing
Drug-efflux upregulation	Intracellular payload concentration decreases	CAR cytotoxicity should not depend on tumour-cell ADC efflux	Human EV cohort and serial case show ABC-transporter associations; ABCG2 directly caused TROP2-retaining SG resistance in UC models	Separate efflux markers from target status in correlative studies
Altered internalisation or trafficking	Impaired ADC uptake or lysosomal processing	Surface-retained antigen could remain CAR-accessible	Sialylation/endocytosis study; general endocytic-evasion literature	Measure surface abundance and ADC trafficking separately
Spatial heterogeneity	Mixed lesion-level exposure and response	Single-target CAR may select antigen-negative lesions	Paired tissue and NECTIN4 PET studies	Sample a relevant progressing lesion; consider multi-site imaging and OR-gated designs
Temporal antigen drift	Archival tissue becomes unreliable	Current CAR eligibility may be misclassified	TURBT–cystectomy–node differences and post-treatment cases	Define a maximum interval and treatment history for eligibility sampling
Alternative target retained	Another ADC/CAR target remains accessible	Target switching or co-targeting may be feasible	TROP2 retained in EV-resistant models; multi-target human IHC	Validate the alternative target in post-treatment tissue before trial assignment
Pharmacologically increased antigen	Higher target abundance may improve ADC and CAR activity	May increase CAR recognition but also normal-tissue liability	PPARγ modulation increased NECTIN4 and CAR activity preclinically	Require tumour/normal tissue pharmacodynamic and safety studies
Immune-excluded target-retaining tumour	Does not necessarily prevent ADC delivery	May limit trafficking, expansion or persistence despite target retention	PPARγ/RXRα activation is associated with CD8+ T-cell exclusion in bladder cancer	Pair target assessment with immune-contexture measures; treat PPARγ as an exploratory contextual marker
Unresolved clinical progression	Resistance mechanism unknown	No reliable inference about CAR susceptibility	Early EV progression cohorts without target assays	Do not infer target loss from radiographic progression alone

Reduced rather than absent antigen creates a more difficult decision. ADC efficacy may sometimes be retained through potent payloads and bystander effects at levels below those required for robust CAR activation, whereas an activated CAR product may amplify weak signals but incur normal-tissue toxicity. CAR structure itself changes the density threshold: costimulatory domain, hinge–transmembrane region, number of immunoreceptor tyrosine-based activation motifs, binder affinity and receptor abundance can all alter sensitivity [33,34]. Iterative engineering has lowered the effective density threshold in a solid-tumour CAR model without sacrificing preclinical safety, underscoring that “antigen-low” is a property of the target–product pair rather than the tumour alone [35]. Because assay platforms and thresholds differ across studies, “NECTIN4-low” cannot currently be translated into a universal CAR exclusion rule. Trials should report the antibody clone, scoring method, membrane restriction, sample timing and quantitative distribution rather than a binary status alone.

### Target-retaining, ADC-specific resistance

The strongest rationale for same-target CAR reuse occurs when the target persists but the ADC fails through downstream pharmacology. In the post-EV biopsy cohort accompanying the NECTIN4 CAR study, expression increased in 9 of 17 matched pairs, while the experimentally resistant RT112 model preserved surface target [3]. A serial molecular-imaging case similarly showed multiple progressive lesions with intense NECTIN4 signal during EV plus pembrolizumab [21]. These results show that progression and target loss are not synonymous.

Drug efflux and payload tolerance are plausible target-retaining mechanisms. In a retrospective cohort of 20 EV-treated patients, higher ABC-transporter expression was associated with poorer response and shorter progression-free survival, and the most favourable group combined NECTIN4 positivity with low transporter expression [9]. These data do not prove that efflux caused resistance, but they show why antigen measurement alone may incompletely explain ADC outcome. A post-ADC CAR trial should therefore distinguish preserved target with a suspected pharmacological resistance mechanism from preserved target with no mechanistic assessment.

### Spatial and temporal heterogeneity

Heterogeneity can make both “target lost” and “target retained” misleading if either conclusion is based on one lesion. First-in-human NECTIN4 PET established the feasibility of non-invasive target visualisation [36]. In ten patients undergoing NECTIN4-targeted PET before EV plus pembrolizumab, uptake varied across lesions within the same patient [37]. Paired tissue cohorts show additional variation by bladder versus upper-tract origin and by primary versus metastatic site [[15], [16], [17]]. Molecular imaging remains investigational and is not a substitute for validated tissue assays, but it exposes a central limitation of single-biopsy eligibility.

For CAR development, spatial heterogeneity can allow antigen-negative clones to progress after same-target therapy, while a negative lesion can obscure antigen retained elsewhere. Protocols should document why the lesion was sampled and whether it was progressing. Whole-body imaging may complement tissue. Serial plasma ctDNA profiling and circulating tumour-cell analysis can track genomic and cellular changes in UC [45,46]. Preliminary plasma cell-free-chromatin analysis has inferred NECTIN4 expression in a small EV-treated cohort, but only at baseline [47]. None establishes CAR-binder-accessible membrane antigen in a progressing lesion; biopsy remains preferred when feasible.

## NECTIN4 after EV: reuse is conditional, not presumptive

NECTIN4 is the anchor example because evidence supports both loss and persistence. NECTIN4 varies by UC subtype [13], while chemotherapy-resistant models and paired clinical specimens show that cytotoxic treatment can accompany downregulation and luminal-to-basal shifts [38]. A 137-pair cohort showed frequent loss of membranous NECTIN4 in metastases, and a separate 47-patient EV cohort linked negative or weak expression to shorter progression-free survival [14]. Later studies confirm variation across bladder resections, cystectomy specimens, lymph nodes and distant metastases [[15], [16], [17]]. Although not all post-EV, these datasets establish that target status is anatomically unstable (Table 1).

The argument for conditional reuse comes from direct resistant-model evidence. EV-resistant RT112 cells retained the target and remained sensitive to two NECTIN4 CAR constructs using different binders, including an enfortumab-derived binder [3]. The latter observation reduces, but does not eliminate, concern that resistance arose through loss of the specific enfortumab-recognised epitope. CAR activity was also retained in resistant xenografts and in a second resistant cell model. When combined with matched post-EV biopsies, these data provide a coherent preclinical proof of principle: if resistance arises downstream of binding and adequate surface antigen persists, changing the effector mechanism can restore activity.

The competing evidence prevents this principle from becoming a blanket sequence. Serial tissue cases demonstrate target reduction during EV, while transporter expression and lesion-level imaging reveal mixed resistance states [21,9,10,32]. The appropriate translational conclusion is therefore conditional:

1. EV progression should trigger a target-state question, not an automatic same-target exclusion.
2. Same-target CAR development is most defensible when a recent progressing lesion retains membranous NECTIN4.
3. Evidence of target loss should redirect development toward another target or an appropriately designed multi-target product.
4. Preserved target without a characterised resistance mechanism remains biologically plausible but uncertain.

This framework also changes how antigen-modulating combinations should be judged. PPARγ agonism increased NECTIN4 and CAR activity preclinically [3], but expanding antigen expression could alter normal-tissue exposure. Any clinical development of pharmacological priming should incorporate paired tumour and normal-tissue pharmacodynamics, rather than assuming that increased target is uniformly favourable.

## Target switching: TROP2 and HER2 are not evidence-equivalent

TROP2 provides the clearest experimental example of target switching after EV. Across four UC cohorts totalling 1,483 localised muscle-invasive tumours, TROP2 was broadly expressed outside the neuroendocrine-like subtype [39]. EV-resistant models lost or reduced NECTIN4 while retaining TROP2 and sacituzumab govitecan activity. Separately, ABCG2-dependent SN-38 efflux caused sacituzumab-govitecan resistance without TROP2 loss, and ABCG2 inhibition restored sensitivity [40]. Resistance can therefore remain target-independent after either a NECTIN4- or TROP2-directed ADC, but neither study links paired human target measurements to a CAR outcome.

Clinical sequencing data also caution against treating target retention as sufficient. In 82 patients receiving sacituzumab govitecan after EV, objective response was 11%, median progression-free survival was 2.1 months and median overall survival was 6.0 months [41]. The cohort was heavily pretreated and was not selected by TROP2 status, so it cannot determine whether target-guided switching would improve outcome. It does show that changing target and payload does not automatically overcome post-EV disease biology.

HER2 is therapeutically important in UC but remains secondary here because its biology, biomarker assessment, ADC trials and resistance have been reviewed recently [23]. The narrower question is whether post-treatment HER2 measurement can support CAR switching or co-targeting after another ADC. Among 2,024 advanced UC samples, 52.5% showed HER2 protein expression, whereas 11.6% had ERBB2 amplification, showing that genomic alteration and protein-defined availability are not interchangeable [42]. In a separate study, ERBB2 status was discordant in 12 of 30 matched primary–metastatic pairs, patient-derived models did not invariably retain source-tumour genotype, and trastuzumab deruxtecan sensitivity depended on more than a simple expression label [43]. These findings support recent, assay-specific reassessment but do not establish HER2 CAR density thresholds. Paired post-ADC evidence is insufficient to place HER2 on the same evidentiary footing as NECTIN4 for this CAR-selection question.

Human co-expression data provide a reason to study switching and co-targeting, but not to assume that either will work. In a 118-specimen UC series, at least one of NECTIN4, HER2 or TROP2 reached an immunohistochemical score of 2+ or higher in 90.7% of specimens and 56.8% showed multi-target co-expression [44]. Larger spatial analyses and rapid-autopsy sampling further demonstrate target heterogeneity, subcellular localisation differences and divergence between primary, nodal and distant sites [17,18]. The matched post-ADC evidence remains sparse. These proportions therefore describe potential target availability in sampled tissue, not the probability that an ADC-exposed patient will qualify for or respond to a multi-target CAR.

UC-specific CAR evidence beyond NECTIN4 remains preclinical. Patient-derived organoids preserved antigen heterogeneity and discriminated MUC1-positive from MUC1-negative responses [48]. SIA-CIgG CAR T cells showed antigen-dependent activity [49], and intravesical mesothelin CAR T cells recognising MUC16 improved tumour control in preclinical models [50]. Patient-derived xenograft and organoid–xenograft systems are research platforms for tracking post-treatment clonal mixtures and CAR activity, but their fidelity, throughput and immune context must be established before patient selection. These studies do not establish clinical efficacy, post-ADC activity or a preferred alternative target.

## From evidence to trial design

The literature does not support a clinical ADC-to-CAR sequence but informs trial design. Enrollment from an archival diagnostic result without checking target status after metastatic evolution and ADC exposure is avoidable. A post-ADC CAR trial should treat target status as dependent on sampling time, intervening therapies and metastatic site [[14], [15], [16], [17]].

### Establish the treatment and resistance context

Prior ADC exposure should be recorded at the level of target, therapeutic binder when known, linker, payload, duration, best response, time to progression and lesion-specific pattern of failure. The target alone is insufficient. Two NECTIN4-directed ADCs may differ in linker and payload, while two ADCs carrying MMAE may share payload resistance despite different antibodies. Likewise, primary progression, acquired progression after response and isolated lesion-level escape represent different biological states. The five-institution EV cohort in which 34 of 121 patients progressed within three months identified clinical features associated with early progression but did not measure the target or resistance mechanism [51]. Such clinical phenotypes can motivate mechanistic hypotheses, but they cannot identify antigen loss or another resistance mechanism.

### Test the most relevant available lesion

When tissue acquisition is feasible within a trial, the preferred specimen is a recent lesion that is clinically relevant to the progression event. A primary resection obtained before systemic therapy is less directly informative for the post-ADC target state than a post-ADC metastatic biopsy. Reports should specify anatomical site, biopsy date, prior ADC details, therapies delivered between sampling and CAR infusion, and whether the sampled lesion was progressing. The substantial discordance across primary, lymph-node and distant metastatic tissue argues for this provenance requirement [[14], [15], [16]].

The assay should be restricted to membrane-accessible antigen and should record antibody clone, staining platform, scoring method, intensity distribution and percentage of positive cells. It should also state whether the diagnostic reagent has demonstrated epitope compatibility with the therapeutic binder; absent such evidence, it should be called an assay-specific target measurement, not a therapeutic-binder assay. A binary positive/negative label discards information that may matter for CAR activation. Where fresh tissue is available, flow cytometry or another quantitative surface assay could complement immunohistochemistry, but no inter-platform conversion or clinically validated threshold exists in UC. Eligibility thresholds should therefore be prespecified as exploratory, justified by the construct’s preclinical activity across defined antigen densities, and retained in analysis even if later found to be poorly discriminative. Supplementary Table S1 provides the proposed reporting fields and distinguishes established target-state measures from contextual markers.

### Use target imaging as a research complement, not a surrogate

NECTIN4-targeted PET can visualise multiple lesions simultaneously and has revealed marked within-patient heterogeneity [37]. Serial imaging has also documented progression with retained signal during EV-based treatment [21]. This capability is conceptually attractive for CAR selection because a biopsy samples only one lesion. However, the first-in-human imaging study and subsequent staging series show that uptake is a composite imaging signal affected by tracer delivery, perfusion, binding and acquisition as well as target abundance [36,37]. No uptake threshold has been validated for CAR recognition. Molecular imaging should currently be used as an exploratory whole-body map linked to tissue and outcome, not as a standalone eligibility test.

### Match the CAR strategy to the resistance state

We propose four investigational decision states.

**State 1: target retained and target-independent resistance supported.** Same-target CAR reuse is most defensible when a recent lesion retains membrane antigen and the resistance phenotype implicates payload tolerance, efflux or another ADC-specific step. The EV-resistant NECTIN4-retaining models directly support this state [3].

**State 2: target retained but resistance unresolved.** Same-target CAR remains biologically plausible, but the trial should describe this as target-informed rather than mechanism-selected enrolment. Correlative studies should test target density, internalisation, efflux and payload-sensitivity hypotheses.

**State 3: target reduced, heterogeneous or uncertain.** A single-target product carries a higher escape risk. Repeat sampling, lesion-level imaging, target switching or an OR-gated multi-target product may be more appropriate for development. This state includes discordant primary and metastatic results and borderline membrane expression.

**State 4: target absent in a sampled progressing lesion.** An unmodified same-target CAR lacks a direct biological rationale. Development should shift to a confirmed alternative target or a construct whose logic does not require the absent antigen. Pharmacological re-expression is experimental and should require tumour/normal-tissue pharmacodynamic and safety evidence.

These states deliberately avoid converting an unvalidated assay threshold into clinical advice. Their purpose is to align the claimed mechanism of a trial with the evidence available for each participant (Fig. 3).

**Fig. 3:**
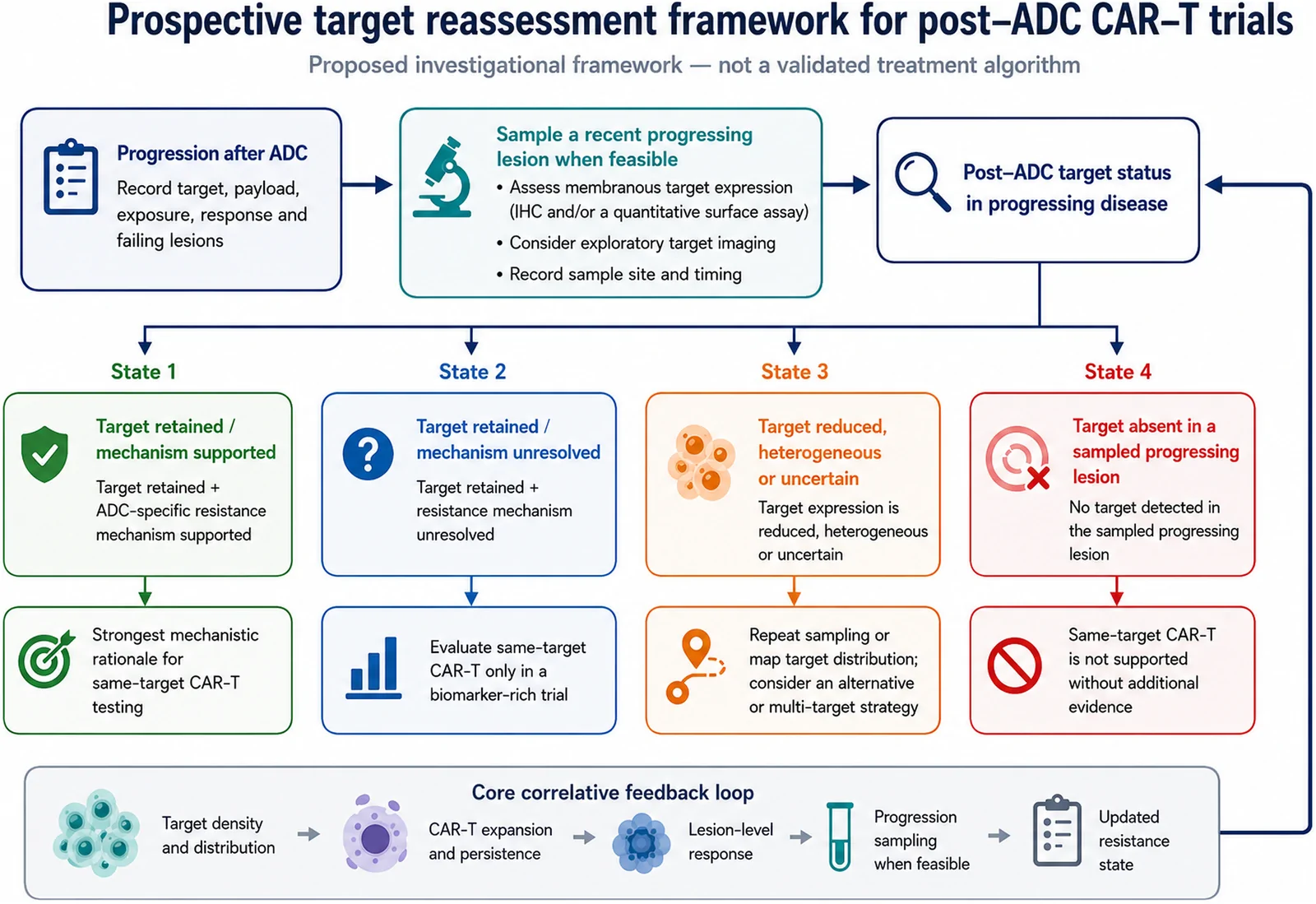
**Prospective target reassessment framework for post-ADC CAR-T trials.** After progression on an ADC, the prior target, payload, exposure, response and failing lesions are documented. A recent progressing lesion is sampled when feasible, with membranous target expression assessed by immunohistochemistry and/or a quantitative surface assay; exploratory target imaging may complement but does not replace tissue assessment, and the sample site and timing are recorded. Four investigational states link retained, unresolved, reduced, heterogeneous or absent target to same-target CAR-T testing, biomarker-rich testing, repeat assessment, alternative-target selection or multi-target development. The core correlative feedback loop connects target density and distribution, CAR-T expansion and persistence, lesion-level response, progression sampling when feasible and an updated resistance state that returns to target reassessment. This is a proposed investigational framework for trial design, not a validated treatment algorithm.

### Engineering can respond to heterogeneity but cannot replace target measurement

OR-gated products can broaden recognition of heterogeneous tumours, whereas AND-gated or sequential circuits seek selectivity by requiring antigen combinations or contexts uncommon in normal tissue. A tandem HER2–IL13Rα2 CAR reduced antigen escape in a heterogeneous solid-tumour model, supporting the engineering principle without providing UC-specific validation [52]. OR gating can widen normal-tissue recognition, while AND gating may exclude tumour cells expressing only one component. Circuit logic should therefore follow lesion-level co-expression and normal-tissue data rather than platform availability [53,54].

Density-sensitive circuits further show why product and biomarker must be co-developed. Greater CAR sensitivity may narrow the safety margin for antigens expressed at low levels in normal tissue [33]. Low-affinity HER2 CAR T cells achieved tumour control without the toxicity observed with higher-affinity constructs in a human-HER2 transgenic model [55], while a synthetic-Notch circuit better distinguished high from low HER2 density than a constitutive CAR [56]. These findings do not validate a UC product, but show that pathology thresholds cannot be separated from binder affinity and circuit design.

For ADC-exposed UC, multi-target development should consequently begin with three linked maps: post-treatment tumour co-expression, inter-lesional discordance and quantitative normal-tissue expression. PPARγ pathway status is a plausible contextual variable because it can couple NECTIN4 regulation to immune exclusion, but it is not a validated criterion for antigen choice or CAR eligibility [3,31]. The available UC tissue series supports the feasibility of studying NECTIN4, TROP2 and HER2 combinations [44], but it does not identify the optimal logic gate. A multi-target product should also be tested against experimentally generated single-positive, double-positive and antigen-low populations, including mixtures that reproduce observed post-treatment heterogeneity. Otherwise, apparent protection from escape may simply reflect testing in uniformly high-expressing models.

### Minimum correlative-study set

Future early-phase studies should report, at minimum:

1. prior ADC target, payload, exposure and lesion-level response;
2. source and timing of the tissue used for CAR target assignment;
3. membrane-specific target score and its distribution;
4. expression of planned alternative or co-targets;
5. product architecture, binder and preclinical density-response relationship;
6. blood expansion and persistence of the CAR product;
7. paired on-treatment or progression tissue when feasible;
8. lesion-specific response linked to baseline target state;
9. assays for plausible ADC-resistance mechanisms, including ABCB1, ABCC1 and ABCG2 where biologically relevant;
10. tumour immune contexture, including evidence of immune exclusion where feasible;
11. normal-tissue and on-target, off-tumour safety observations.

Without these elements, a negative trial could not distinguish target failure, product failure, trafficking failure or immune suppression, and a positive trial could not establish why target reuse succeeded.

## Discussion: evidence limitations and research priorities

The main gap is clinical: no published UC-specific cohort has reported CAR-engineered-cell outcomes after prior ADC exposure. Direct evidence rests on resistant cell lines, immunodeficient xenografts and a small post-EV biopsy cohort [3]. These data establish plausibility, not efficacy or safety. The broader UC CAR literature is predominantly preclinical [48,49], and normal-tissue expression constrains several candidate antigens [12].

A second limitation is that “post-treatment” evidence is heterogeneous. Some of the largest paired cohorts compare untreated primary tumours with metastases obtained before metastatic therapy [14]. They establish spatial and evolutionary instability but do not estimate the effect of EV. Conversely, serial reports during EV are often single cases [21,10,32]. These evidence types answer different questions and should not be pooled into a single prevalence estimate.

Third, available assays were developed mainly for pathology description or ADC research, not CAR selection. H-score thresholds associated with EV outcomes cannot be assumed to predict CAR activation, and transcript abundance cannot substitute for accessible epitope density. The central practical gap is not simply lack of a numerical cut-off: publications variably report the antibody clone, epitope relation to the therapeutic binder, platform, membrane restriction and scoring rule. Prospective studies should compare membrane immunohistochemistry, quantitative surface assays and target imaging against product-specific functional thresholds, using the reporting framework in Supplementary Table S1.

Fourth, target switching has stronger biological than clinical support. TROP2 remained expressed and sacituzumab govitecan retained potency in EV-resistant models [39], yet post-EV activity was modest in an unselected, heavily pretreated cohort [41]. Antigen availability is only one component of sensitivity. HER2 has an expanding ADC evidence base, but post-ADC tissue linked to CAR function remains sparse. Multi-target engineering adds construct-specific uncertainty and is not a generic correction for heterogeneous staining.

Six priorities follow from these limitations:

1. prospective paired biopsies before ADC and at defined resistance;
2. standardised membrane-antigen quantification linked to resistant phenotype;
3. patient-derived models that preserve post-ADC clonal heterogeneity;
4. direct comparison of ADC and CAR activity in the same resistant model;
5. biomarker-rich early-phase trials stratified by prior ADC and recent target state;
6. assay-comparison studies that link clone, epitope compatibility, surface density and CAR-product function.

An informative study would enroll an ADC-exposed UC cohort, embed recent target assessment when feasible, prespecify resistance states and connect lesion-level response to antigen density and mechanism. It could determine whether treatment history predicts response to cellular therapy rather than merely describing the cohort.

## Conclusions

ADC exposure turns CAR target selection in UC from a static expression problem into a dynamic resistance-state problem. Across human tissue, serial imaging and resistant models, membranous NECTIN4 may be lost or retained; retention can coexist with EV-specific resistance downstream of target binding. In that setting, preclinical evidence indicates that changing the effector platform from an MMAE-containing ADC to a NECTIN4 CAR can restore activity. TROP2 and HER2 may provide switching or co-targeting options, although their post-treatment CAR evidence is less direct.

ADC progression is therefore not a biomarker of antigen loss and should not, by itself, determine same-target reuse. Within trials, progression should trigger reassessment of accessible target in a relevant lesion and evaluation of whether failure is antigen-dependent. Until clinical outcome data mature, this framework is a trial-development tool rather than a treatment algorithm. Its value is to make positive and negative CAR trials mechanistically interpretable while avoiding both futile target reuse and premature abandonment of a biologically intact target.

## Glossary

**Accessible membrane antigen:** Target epitope exposed on the tumour-cell surface and available for binding by an ADC or CAR.

**ADC:** Antibody-drug conjugate; a target-binding antibody linked to a cytotoxic payload.

**CAR:** Chimeric antigen receptor; a synthetic receptor that redirects an immune cell to a selected surface antigen.

**EV:** Enfortumab vedotin, a NECTIN4-directed ADC carrying monomethyl auristatin E.

**MMAE:** Monomethyl auristatin E, a microtubule-disrupting ADC payload.

**Resistance state:** The target and mechanism context underlying progression after ADC exposure.

**Same-target CAR reuse:** Development of a CAR against the antigen targeted by the preceding ADC.

**Target switching:** Selection of a different surface antigen after loss, reduction or heterogeneity of the prior target.

## Data availability

No new patient-level or experimental dataset was generated for this review. Search strategies, screening documentation and derived evidence tables are supplied as supplementary files. Bibliographic records remain subject to the terms of their source databases.

## Ethics approval and consent

Not applicable. This article reviews previously published studies and contains no new research involving human participants or animals.

## Declaration of generative AI and AI-assisted technologies in the manuscript preparation process

During the preparation of this work, the authors used OpenAI Codex for document organisation, search-strategy development and language editing. After using this tool, the authors reviewed and edited the content as needed and take full responsibility for the content of the published article. All study selections, evidence extractions, interpretations and references were verified by the authors against primary sources.

## CRediT authorship contribution statement

**Jiayi Liu:** Writing – review & editing, Writing – original draft, Visualization, Project administration, Methodology, Investigation, Funding acquisition, Formal analysis, Data curation, Conceptualization. **Shaoqiang Xing:** Writing – review & editing, Validation, Investigation. **Qian Liu:** Writing – review & editing, Validation, Supervision, Project administration, Methodology, Funding acquisition, Conceptualization.

## Declaration of competing interest

The authors declare that they have no known competing financial interests or personal relationships that could have appeared to influence the work reported in this paper.

## Appendix Supplementary materials

**Supplementary File 1:** Supplementary methods and search documentation, including the review question, eligibility framework, database searches, screening workflow, evidence hierarchy and synthesis boundaries. **Supplementary File 2:** Derived evidence documentation workbook containing the executed PubMed search log, database-export audit, Embase batch audit, claim-changing manual decisions, included-study shortlist, structured core-evidence extraction, citation-verification log and claim–evidence map. Licensed raw bibliographic exports are not redistributed. **Supplementary Table S1:** Assay-reporting framework and marker maturity for post-ADC CAR target assessment in urothelial carcinoma.
