# Supplementary material for: Antigen persistence after antibody-drug conjugates in urothelial carcinoma: implications for CAR-cell target selection
Source: Neoplasia. 2026 Oct 17;82:101383. doi: 10.1016/j.neo.2026.101383 (PMC13718832; doi:10.1016/j.neo.2026.101383)
Supplement: Supplementary file 1 [file mmc1.docx]

Supplementary methods and search documentation

# Review designation

This article is a critical review supported by a reproducible, systematic evidence search. It is not presented as a completed systematic review or meta-analysis. The search and screening process was designed to identify the evidence needed to evaluate post-antibody–drug conjugate (ADC) target states and their implications for chimeric antigen receptor (CAR)-cell development in urothelial carcinoma (UC).

# Review question

The review asked whether resistance to a target-directed ADC in UC reflects loss of the corresponding surface antigen or a target-independent mechanism, and how that distinction should influence same-target CAR reuse, target switching, multi-target engineering and trial eligibility testing.

# Eligibility

Eligible evidence comprised:

1. human UC studies with paired, serial, metastatic or post-treatment tissue;

2. mechanistic studies of resistance to NECTIN4-, HER2- or TROP2-directed ADCs in UC;

3. UC-specific CAR-cell studies targeting ADC-associated or alternative surface antigens;

4. studies informing quantitative antigen measurement, target modulation or multi-target CAR design; and

5. cross-tumour mechanistic studies only when the relevant principle was not adequately studied in UC and the limits of transferability could be stated.

Treatment-naive bulk RNA studies without surface or protein validation, purely computational rankings, efficacy-only ADC reports without target-state information and non-UC CAR studies without a transferable mechanism were excluded from the core evidence synthesis.

# Information sources

MEDLINE/PubMed was searched on 26 July 2026 through the NCBI E-utilities interface. Embase, Scopus and Web of Science Core Collection were searched on 27 July 2026 through institutional web access. OpenAlex was searched in a final expansion pass using four concept families: UC–EV resistance–NECTIN4–CAR; general ADC resistance; CAR antigen density/heterogeneity/engineering; and UC CAR/organoid/NECTIN4 expression. Crossref, publisher records and PubMed Central were used for bibliographic or full-text verification. Citation chasing from decisive primary studies and recent competing reviews was used to identify older mechanistic work.

# Executed PubMed search families

Eight searches were run without language or publication-date filters:

| **ID** | **Focus** | **Records returned** |
| --- | --- | --- |
| Q1 | NECTIN4/enfortumab target state and resistance | 344 |
| Q2 | HER2/disitamab target state and resistance | 404 |
| Q3 | TROP2/sacituzumab target state and resistance | 152 |
| Q4 | Direct ADC–CAR intersection in UC | 11 |
| Q5 | ADC-resistance mechanisms in UC | 192 |
| Q6 | Target reassessment by tissue or imaging | 404 |
| Q7 | CAR antigen escape and multi-target engineering | 8 |
| Q8 | Overlapping UC CAR reviews | 31 |

Because individual articles could be returned by several searches, these counts must not be summed as unique records. After automated deduplication by PMID, DOI and normalised title, the candidate file contained 1,098 unique records.

The exact machine-executed query strings, dates, returned counts and raw-file identifiers are supplied in ctr_pubmed_search_log.tsv.

# Subscription-database expansion

The subscription-database searches used aligned concept blocks for:

1. disease: urothelial, bladder, transitional-cell or urinary-tract cancer;

2. platform or target: chimeric antigen receptor/CAR T/CAR NK, ADC, enfortumab, NECTIN4, HER2/ERBB2, TROP2/TACSTD2, sacituzumab or disitamab; and

3. decision mechanism: resistance, antigen loss, density, expression, heterogeneity, discordance, paired/serial sampling, organoid or target reassessment.

In Scopus, the query was run in Article title, Abstract and Keywords and returned 1,190 records. In Web of Science Core Collection, the equivalent Topic query returned 935 records. Embase returned 1,793 records, exported in four complete batches of 500, 500, 500 and 293. The Embase RIS files did not retain a verbatim search-history string; the concept blocks and field restriction above are therefore reported rather than an unverified reconstruction of interface syntax.

The subscription-database query was deliberately sensitivity-oriented. An audit of the Embase export found 1,300 records without both a UC disease concept and a relevant CAR/ADC/target concept in title, abstract or keywords; these were treated as retrieval noise. The remaining 493 Embase records entered focused title/abstract triage. The four database exports contained 5,016 source records in total and 3,601 unique candidates after DOI, PMID and normalised title–first-author deduplication. Database presence alone was not an inclusion criterion.

# Screening and prioritisation

Candidate records were prioritised using title, abstract, publication type, query provenance and the presence of post-treatment, target-state, resistance or CAR-engineering concepts. The initial PubMed workflow produced:

- 1,098 unique candidate records;
- 116 records in the priority-screening file;
- 30 records in the core/competing-review shortlist; and
- 41 references directly cited before subscription-database expansion.

The Embase/Scopus/Web of Science expansion added four claim-changing primary studies to the cited synthesis: TROP2-retaining, ABCG2-mediated sacituzumab-govitecan resistance; standardized HER2 protein and ERBB2-amplification assessment; genomic and model-level HER2 discordance with functional ADC testing; and intravesical MUC16-directed CAR T-cell evidence. The final manuscript cites 45 identifier-verified references. Lower-directness expression surveys, efficacy-only ADC reports, redundant reviews and meeting abstracts were retained in the candidate archive but not added merely to increase the reference count.

Jiayi Liu performed the primary screen. Shaoqiang Xing independently verified all included studies and a sample of exclusions, with disagreements resolved by consensus with Qian Liu.

The counts above describe an evidence-development workflow rather than a PRISMA study-selection flow. The final cited set was expanded after the priority screen by subscription-database searching, targeted OpenAlex searching, backward citation chasing and PMID-level verification. Automated concept triage was used to manage a deliberately broad retrieval set, and all records proposed for citation underwent manual title/abstract and claim-level verification.

# Data extraction

Extracted fields included bibliographic identifiers; disease and treatment context; target and assay; specimen timing and anatomical site; paired or unpaired design; antigen result; resistance mechanism; CAR platform and model; directness for the review question; principal supported claim; major limitations; and full-text verification status.

Evidence was ordered by directness:

1. paired or serial human post-ADC tissue with clinical context;

2. paired primary–metastatic human tissue;

3. unpaired post-ADC human tissue;

4. ADC-resistant patient-derived models with CAR testing;

5. resistant cell-line or xenograft models;

6. treatment-naive human tissue;

7. treatment-naive organoid or animal models;

8. cell-line-only studies; and

9. cross-tumour mechanistic extrapolation.

This hierarchy indicates relevance to the review question and is not a formal risk-of-bias instrument.

# Synthesis

No pooled efficacy estimate was planned. Evidence was grouped by resistance mechanism and its decision consequence. Human and preclinical evidence were kept separate, contradictions were retained, and differences in assay, anatomical site, treatment timing and model were used to explain discordant findings.

Scenarios were classified as:

- same-target CAR biologically plausible;
- target retesting required;
- target switching or multi-target CAR preferred for trial development; or
- insufficient evidence.

These categories are investigational trial-development judgments, not clinical treatment recommendations.

# Search and reporting limitations

The evidence base is limited by sparse paired post-ADC human tissue, no established CAR eligibility threshold for NECTIN4/HER2/TROP2, reliance on preclinical CAR evidence and a sensitivity-oriented subscription-database retrieval that required automated concept triage. Embase did not preserve the verbatim interface query in the RIS exports. Accordingly, the article is described as a critical review with a reproducible multi-database search and does not claim formal systematic-review or PRISMA compliance.

# Supplementary source files

- ctr_pubmed_search_log.tsv: exact executed PubMed searches.
- expanded_multisource_search_2026-07-26.tsv: final OpenAlex expansion queries and retrieval record.
- database_export_audit_2026-07-27.md: PubMed, Scopus, Web of Science and Embase export integrity and deduplication audit.
- embase_export_batch_audit_2026-07-27.tsv: Embase batch counts, accession continuity and automated concept triage.
- multidatabase_deduplicated_candidates_2026-07-27.tsv: harmonized four-database candidate archive with provenance.
- multidatabase_manual_decisions_2026-07-27.tsv: manual decisions for the claim-changing new evidence and key exclusions.
- ctr_pubmed_candidates.tsv: deduplicated candidate set.
- priority_screening.tsv: prioritised records with working decisions.
- included_study_shortlist.tsv: core and competing-review shortlist.
- core_evidence_extraction.tsv: structured extraction of decisive evidence.
- citation_verification.tsv: identifier and citation verification.
- claim_evidence_map.tsv: mapping from manuscript claims to evidence and claim limits.
