# Supplementary material for: Antigen persistence after antibody-drug conjugates in urothelial carcinoma: implications for CAR-cell target selection
Source: Neoplasia. 2026 Oct 17;82:101383. doi: 10.1016/j.neo.2026.101383 (PMC13718832; doi:10.1016/j.neo.2026.101383)
Supplement: Supplementary file 2 [file mmc2.docx]

**Supplementary Table S1. Assay-reporting framework and marker maturity for post-ADC CAR target assessment in urothelial carcinoma**

| **Measurement or marker** | **What it can support** | **Required reporting fields** | **What it cannot establish alone** | **Current maturity for post-ADC CAR development** |
| --- | --- | --- | --- | --- |
| Membrane-focused target IHC (NECTIN4, TROP2, HER2 or candidate alternative) | Tissue-level target presence, distribution and heterogeneity in the sampled lesion | Antibody clone/vendor; platform; fixation; membrane-versus-cytoplasmic scoring rule; intensity and percentage distribution; H-score or prespecified score; specimen site/date; intervening therapy | Recognition of the therapeutic antibody or CAR binder unless epitope compatibility has been demonstrated; a product-specific CAR density threshold | Essential trial-enrolment component when tissue is available, but not yet standardised for CAR eligibility |
| Quantitative fresh-tissue surface assay (e.g., flow cytometry) | Relative surface abundance on viable tumour cells; intralesional distribution; possible link to product-specific functional assay | Dissociation method; tumour-cell gate; reagent and epitope; calibration material; viable-cell fraction; distribution rather than binary result | Whole-body heterogeneity; direct conversion to IHC score without validation | High biological directness; exploratory because fresh metastatic tissue is not always feasible |
| Therapeutic-binder or CAR-binder assay | Whether the intended therapeutic recognition element binds the measured tumour cells | Binder identity; epitope/competition evidence; affinity format; assay platform; positive/negative controls; relation to final clinical construct | Clinical efficacy or safety | Preferred research-grade bridge between pathology and the final product; rarely available in routine practice |
| NECTIN4-targeted PET | Whole-body lesion map and inter-lesional uptake heterogeneity | Tracer; acquisition/reconstruction; lesion-selection rule; timing relative to therapy; uptake metric; paired tissue when available | A validated CAR activation threshold or replacement for biopsy | Exploratory complement to tissue; no validated CAR-selection cutoff |
| DNA alteration (e.g., NECTIN4 or ERBB2 amplification) | Contextual enrichment information; in some settings association with protein expression or ADC outcome | Assay; copy-number threshold; tumour fraction; specimen provenance; paired protein result | Accessible surface epitope, target distribution or CAR recognition | Contextual only; not a stand-alone CAR-selection biomarker |
| RNA expression or molecular subtype | Contextual target biology, subtype association and longitudinal hypothesis generation | Platform; normalisation; tumour cellularity; subtype classifier; specimen provenance; paired surface-protein result | Surface density, therapeutic-binder compatibility or CAR eligibility | Contextual only; should be interpreted with a membrane assay |
| ABCB1/P-glycoprotein, ABCC1/MRP1 and ABCG2/BCRP | Hypothesis that ADC payload efflux contributes to resistance while extracellular target is retained | Assay and cellular localisation; target measurement in the same lesion; ADC payload; timing relative to progression | Causality in an individual patient or CAR resistance | Exploratory resistance correlates |
| PPARγ/RXRα pathway activity and immune-contexture markers | Potential coupling of target regulation, immune exclusion and microenvironmental resistance | Genomic/transcript/protein method; CD8 and stromal measures; specimen provenance; paired target result | A validated criterion for CAR target selection or pharmacological priming | Exploratory contextual markers |
| Circulating tumour DNA, circulating tumour cells or cell-free-chromatin expression inference | Repeated, low-burden longitudinal sampling and hypothesis generation | Analyte; collection time; analytical sensitivity; lesion/tissue comparator; preplanned interpretation | Product-relevant membrane-antigen density or a substitute for a progressing-lesion biopsy | Exploratory complement only |

Interpretation notes. The framework is deliberately product aware. A pathology assay may provide useful evidence of target state while still failing to show recognition by the therapeutic binder. For every assay, the lesion source and timing relative to the preceding ADC are part of the result. “Essential” denotes an evidence need for interpretable trial enrolment, not a validated clinical test.
